# Rapid detection of Yunnan Xiaomila based on lightweight YOLOv7 algorithm

**DOI:** 10.3389/fpls.2023.1200144

**Published:** 2023-06-05

**Authors:** Fenghua Wang, Jin Jiang, Yu Chen, Zhexing Sun, Yuan Tang, Qinghui Lai, Hailong Zhu

**Affiliations:** ^1^ Faculty of Modern Agricultural Engineering, Kunming University of Science and Technology, Kunming, Yunnan, China; ^2^ Engineering Training Center, Kunming University of Science and Technology, Kunming, Yunnan, China

**Keywords:** improved YOLOv7, *Capsicum frutescens L.*, detection, unstructured environment, lightweight

## Abstract

**Introduction:**

Real-time fruit detection is a prerequisite for using the Xiaomila pepper harvesting robot in the harvesting process.

**Methods:**

To reduce the computational cost of the model and improve its accuracy in detecting dense distributions and occluded Xiaomila objects, this paper adopts YOLOv7-tiny as the transfer learning model for the field detection of Xiaomila, collects images of immature and mature Xiaomila fruits under different lighting conditions, and proposes an effective model called YOLOv7-PD. Firstly, the main feature extraction network is fused with deformable convolution by replacing the traditional convolution module in the YOLOv7-tiny main network and the ELAN module with deformable convolution, which reduces network parameters while improving the detection accuracy of multi-scale Xiaomila targets. Secondly, the SE (Squeeze-and-Excitation) attention mechanism is introduced into the reconstructed main feature extraction network to improve its ability to extract key features of Xiaomila in complex environments, realizing multi-scale Xiaomila fruit detection. The effectiveness of the proposed method is verified through ablation experiments under different lighting conditions and model comparison experiments.

**Results:**

The experimental results indicate that YOLOv7-PD achieves higher detection performance than other single-stage detection models. Through these improvements, YOLOv7-PD achieves a mAP (mean Average Precision) of 90.3%, which is 2.2%, 3.6%, and 5.5% higher than that of the original YOLOv7-tiny, YOLOv5s, and Mobilenetv3 models, respectively, the model size is reduced from 12.7 MB to 12.1 MB, and the model’s unit time computation is reduced from 13.1 GFlops to 10.3 GFlops.

**Discussion:**

The results shows that compared to existing models, this model is more effective in detecting Xiaomila fruits in images, and the computational complexity of the model is smaller.

## 1 Introduction

As one of the important vegetable crops, chili pepper has the highest output value and benefit (Ye et al., 2021). The special chili pepper industry has become the pillar industry for agricultural and rural economic development in some areas of Yunnan (Ren et al., 2022).Currently, the research on chili pepper harvesting machinery in China mainly focuses on the one-time harvesting of bell peppers, linear peppers, and other chili pepper varieties (Zhang and Xu, 2019; Su et al., 2021; Yuan et al., 2021). However, Xiaomila peppers in Yunnan are harvested in batches during the flowering and fruiting periods, and traditional mechanical one-time harvesting methods cannot adjust to the characteristics of Xiaomila pepper picking (Zhu et al., 2022).

With the advent of agricultural digitization 4.0 (Abbasi et al., 2022), advanced sensor technology, the Internet of Things (IoT), and artificial intelligence (AI) are widely used for fruit detection, information collection, and fruit analysis in agriculture (Chamara et al., 2022), and agricultural picking robots have entered the public’s vision. The rapid and accurate detection of ripe fruits has become a research hot spot (Lv et al., 2022). The green and ripe fruit of Xiaomila has a light yellow-green peel, and smooth, or slightly wrinkled skin, and a single plant has a high fruit-bearing rate, with irregularly distributed space, making it difficult to detect Xiaomila with an embedded device in the orchard or field environments. Therefore, it is very necessary to carry out research on lightweight target detection methods for crop fruits with dense targets, small sizes, and high occlusion.

At present, two methods are mainly used for fruit target detection. One is the traditional image detection and segmentation technology that mainly uses color (Janani and Jebakumar, 2023; Tajdar et al., 2023), texture (Alshehhi and Marpu, 2023; Chapeta et al., 2023), edge (Xie et al., 2022; Quan et al., 2023), and other feature information. However, the shallow features can only detect the target in a limited scene, and these methods often lack generalizability and robustness.

As deep learning network has been widely applied to crop target detection (Fu et al., 2022), researchers began to use deep learning networks to solve crop detection problems in complex environments. For example, Iqbal and Hakim (2022) proposed a deep learning-based method for automatic classification and grading of eight harvested mango varieties using Inception v3, considering features such as color, size, shape, and texture. The proposed approach achieved up to 99.2% classification accuracy and 96.7% grading accuracy. However, this study was conducted under a single background condition and did not consider the impact of complex background conditions in non-structured environments on recognition. Zhou et al. (2023) modified the YOLOv7 model to detect Camellia oleifera fruits and determine the center point of the fruit recognition frame. Image processing and a geometric algorithm were used to process the image, segment the fruit, determine its morphology, extract the centroid of the fruit’s outline, and analyze the position deviation between its centroid point and the center point in the YOLO recognition frame. Accurate detection results were achieved for Camellia oleifera fruits under different lighting conditions and when the fruits were occluded. Tang et al. (2023a) developed a fruit detection model based on the YOLOv4-tiny architecture. The proposed method utilizes the generated bounding boxes from the model to extract the regions of interest for fruits. Subsequently, an adaptive stereo matching is performed based on the bounding box generation mechanism. The model demonstrates robust fruit detection under various lighting conditions. However, these studies are specifically focused on regular-shaped Camellia oleifera fruits and may not be applicable to irregularly growing Xiaomila peppers with varying growth directions. Wang et al., (2022) modified the YOLOv5s model (YOLOv5sCFL) by replacing the Conv layer in the cross-stage part with GhostConv and adding a coordinated attention (CA) layer and using a bidirectional feature pyramid Network (BiFPN) to replace the PANet (path aggregation network) in the neck to improve detection accuracy. While this study improved the computational speed of the model, it did not achieve significant improvements in terms of detection accuracy and model size. Wu et al. (2022) proposed a fruit detection method by using the YOLOv7 network with multi-data augmentation for detecting fruits in complex field scenes. The proposed method effectively improves the model’s generalization capability. However, it did not take into account factors such as model size and runtime speed. Zhong et al. (2022) proposed an improved fast R-CNN algorithm for the small size and cluster growth of pepper fruits in the detection process, which effectively improved the ability to extract small features. Cong et al. (2023) proposed an improved Mask RCNN with the Swin Transformer attention mechanism and exploited UNet3+ to improve the mask head and mask segmentation quality to efficiently segment sweet peppers of different categories under leaf occlusion. As representatives of two-stage object detection algorithms, although the R-CNN series algorithms have high detection accuracy, their running speed and model parameter size are difficult to meet the requirements of real-time detection and embedded development in agricultural applications. Li et al. (2021) combined the idea of multi-scale prediction and attention mechanism with the YOLOv4-tiny backbone to improve the recognition performance of occluded and small bell peppers. Nan et al. (2023) used NSGA II to prune the YOLOv5l model and obtained a lightweight bell pepper detection model. Although both of these models have achieved high accuracy in bell pepper detection, it is important to note that the study was conducted in orchards and did not consider various factors in unstructured environments, such as lighting, that may affect the accuracy of detection.The above research shows that deep learning algorithms such as YOLO have become the mainstream fruit detection methods, and this type of algorithm has been improved in different ways to improve its target detection effect in unstructured environments. However, the slow running speed of the network, the large network weight file, and the low detection accuracy of the network for multi-scale alternating targets and occluded targets are still problems that need to be solved urgently (Tang et al., 2023b). To solve these problems, this paper designs an improved YOLOv7-tiny model. The contributions of the model proposed in this article can be summarized as:

1) We propose a lightweight one-stage detection model based on YOLOv7, called YOLOv7-PD, for real-time detection of Xiaomila fruits. Deformable convolutions are used to significantly reduce FLOPS and model weight size, while SE modules are used to enhance the feature extraction capabilities of the network.

2) We improve the network’s detection performance of complex poses of Xiaomila fruits by applying techniques such as horizontal flipping and random rotation to the original images. We also add noise and adjust image brightness to reduce the inconsistencies in brightness caused by different light intensities and visual sensor differences, in order to improve clarity. Furthermore, we increase the number of targets in the images by mosaic stitching, which enhances the detection performance of densely-packed targets.

3) We have determined the effectiveness of the model through ablation experiments and model comparison experiments. Among various fruit detection models, the model proposed by us achieved the highest accuracy and required the least number of FLOPS and computational resources.

## 2 Materials and methods

### 2.1 Collection of a Xiaomila fruit dataset

**Figure 1:**
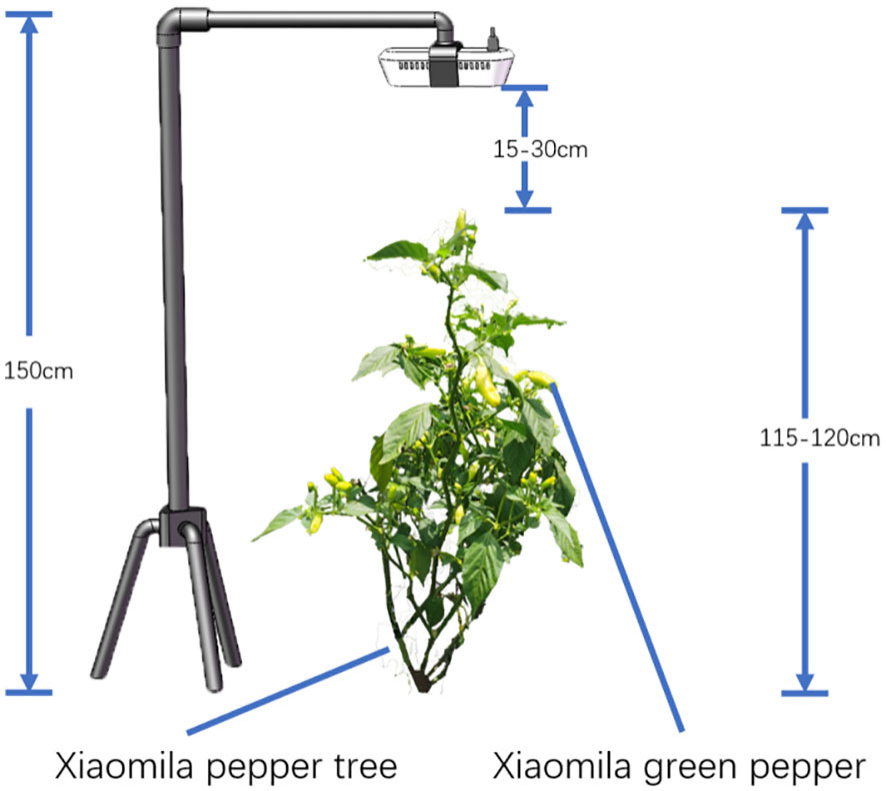
The schematic diagram of the Xiaomila collection method.

**Figure 2:**
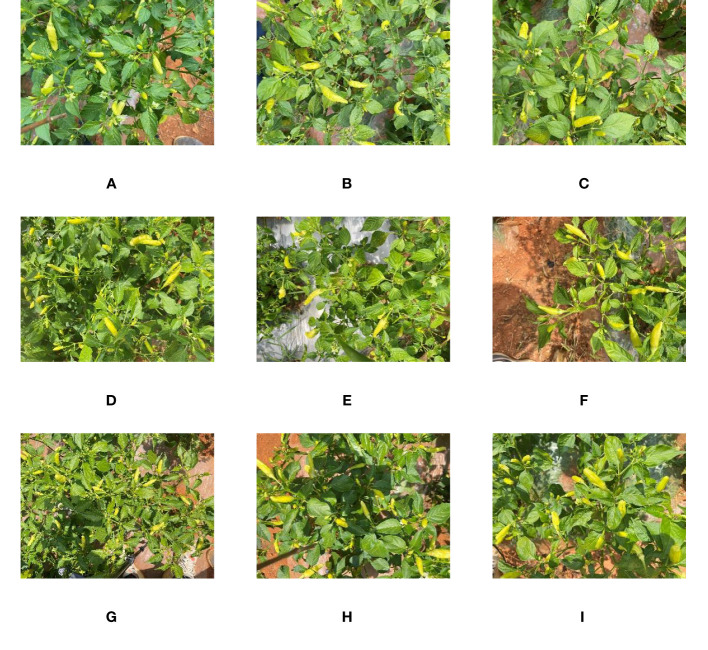
An example of Xiaomila data collection: **(A-C)** backlight; **(D-F)** weak light intensity; **(G-I)** strong light intensity.

On August 2, 2022, at Shupi Village, Yi Nationality Township, Qiubei County, Wenshan Autonomous Prefecture, Yunnan Province (104° 6′ 44″ N, 23° 53′ 7″ E), Yunxiao Lai No.10 was taken as the research object. Under different natural lighting conditions, the Intel RealSense D435i camera was placed 15 to 30 cm directly above the Xiaomila pepper plant, and the RGB images of the Xiaomila pepper in the mature stage were collected. The resolution of the image was 1920×1080 pixels, and a total of 1500 images were collected. The schematic diagram of the picking method and the collected images are shown in **Figures 1**, **2**.

### 2.2 Production of a Xiaomila dataset

**Figure 3:**
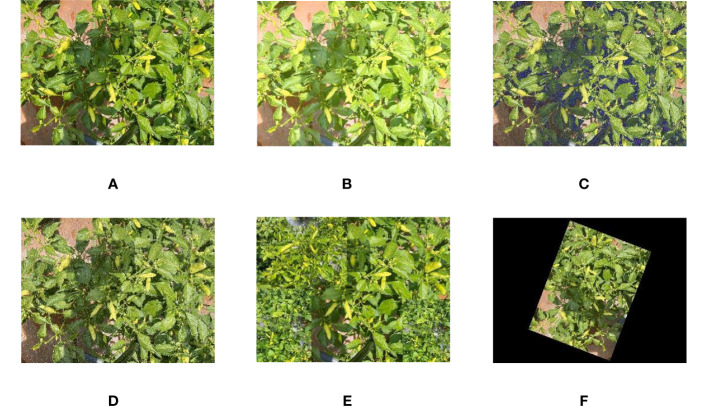
An example of Xiaomila image enhancement: **(A)** original image **(B)** adjusting brightness **(C)** adding Gaussian noise **(D)** adding salt and pepper noise **(E)** mosaic stitching **(F)** random angle rotation.

Considering the impact of the complex environment on fruit detection in the Xiaomila picking process, to avoid model training overfitting and improve the robustness of the model, the original image was enhanced (Akbar et al., 2022; Shoaib et al., 2022; Bosquet et al., 2023) through image mirroring, random rotation, and other methods to improve the detection effect of the network for Xiaomila fruits with complex postures. By adding noise and adjusting image brightness, image brightness deviations caused by different light intensities and differences in visual sensors were reduced. Then, the number of objects in the image was increased through mosaic stitching, thus improving the detection performance of dense objects. The data enhancement method is shown in **Figure 3**. In this way, the dataset was expanded to 4000 images, and the expanded images were manually marked in the YOLO format using Labelimg software. Then, the dataset was divided into a training set, a test set, and a verification set at a ratio of 7: 2: 1 (the training set is used to train the network parameters, the test set is used to test the generalization ability of the model after training, and the verification set is used to tune the hyperparameters used in the model training process to improve the model performance). Besides, to ensure the reliability of the trained model, duplicated images between datasets were removed.

### 2.3 Construction of the Xiaomila target detection model

#### 2.3.1 YOLOv7 model

**Figure 4:**
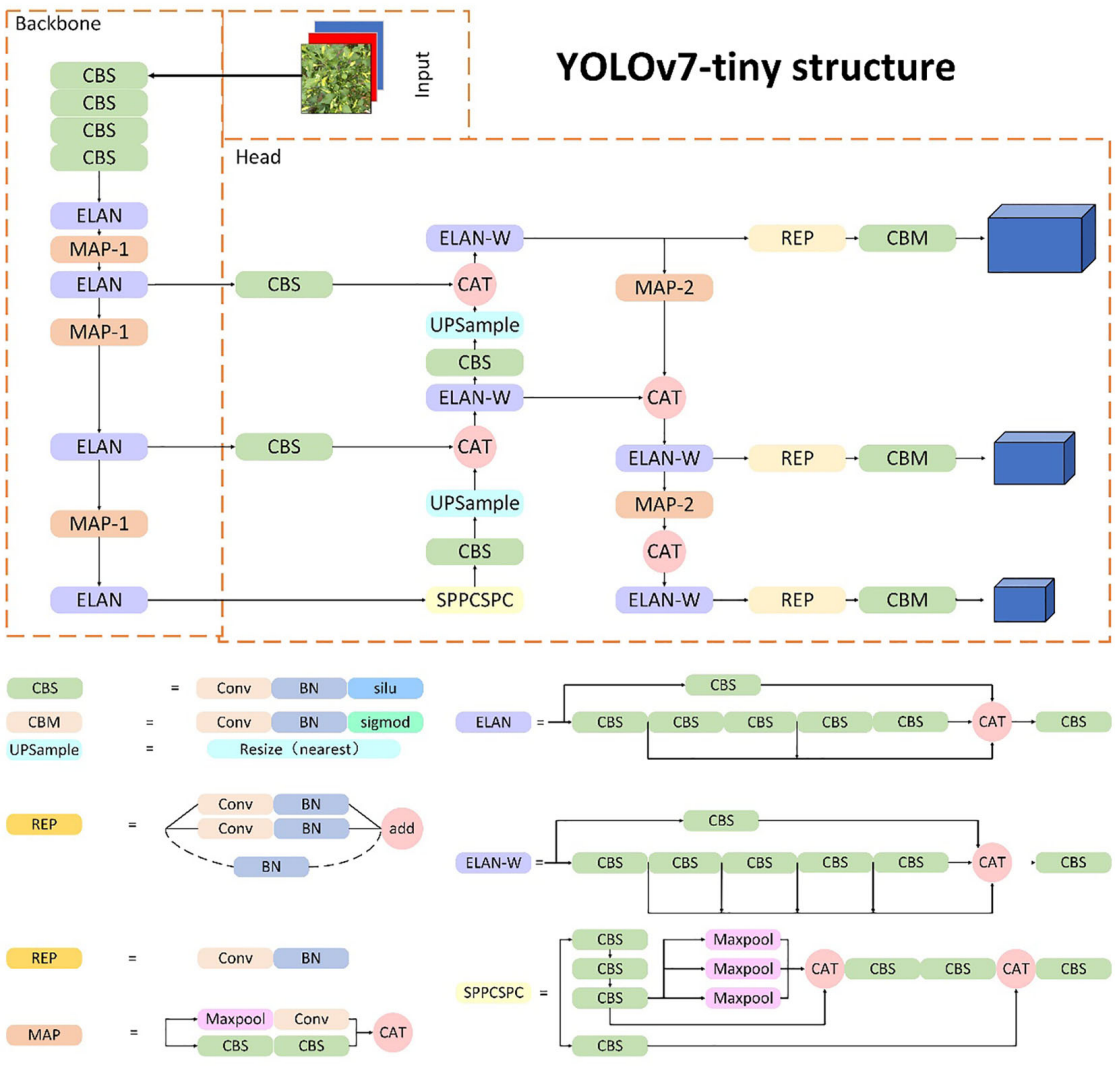
The architecture of the YOLOv7 network.

The YOLOv7 model is an anchor-based target detection algorithm, which can achieve a fast detection speed while maintaining high accuracy. It has seven versions, namely, YOLOv7, YOLOv7-d6, YOLOv7-e6, YOLOv7-e6e, YOLOv7-tiny, YOLOv7x, and YOLOv-w6, to meet the needs of different application scenarios and computing resources (Wang et al., 2022). As shown in **Figure 4**, the YOLOv7-tiny framework consists of three parts: backbone, neck, and head. The backbone part is mainly constructed by convolution, the E-ELAN (Extended-ELAN) module, the MPConv (Max Pooling Conv) module, and the SPPCSPC module. Specifically, based on the original ELAN (Zhang et al., 2022), the E-ELAN module changes the calculation block while maintaining the transition layer structure of the original ELAN, and it enhances the ability of network learning by expanding, shuffling, and merging cardinality. The MPConv module uses parameters of different precisions for convolutional operations to trade off between computational complexity and accuracy. The SPPCSPC module is used to enhance the expressive power of convolutional neural networks. It is composed of two modules: the spatial pyramid pooling (SPP) module and the cross-stage partial network (CSP) module. The SPP module is designed for multi-scale object detection and classification tasks. It partitions the input feature map into multiple sub-regions by adding a pooling layer to the network and pools each sub-region to obtain a fixed-size feature vector. The CSP module is used to reduce network parameters and computational complexity. It divides the network into two parts: one for feature extraction and the other for feature processing and fusion, thus reducing the number of parameters and computations in the network. The combination of the SPP module and CSP module in the SPPCSPC module can improve the network’s expressive power and computational efficiency simultaneously. The neck module is used to combine feature maps at different levels to generate feature maps with multi-scale information to improve object detection accuracy. The head network takes the multi-scale feature maps generated by the neck network and performs object detection. The head network uses anchor boxes to predict the location, size, and class of objects in the input image. The predicted object boxes are then refined by a post-processing step called Non-Maximum Suppression (NMS) to eliminate redundant detections and improve the model’s precision.

#### 2.3.2 Model improvement

**Figure 5:**
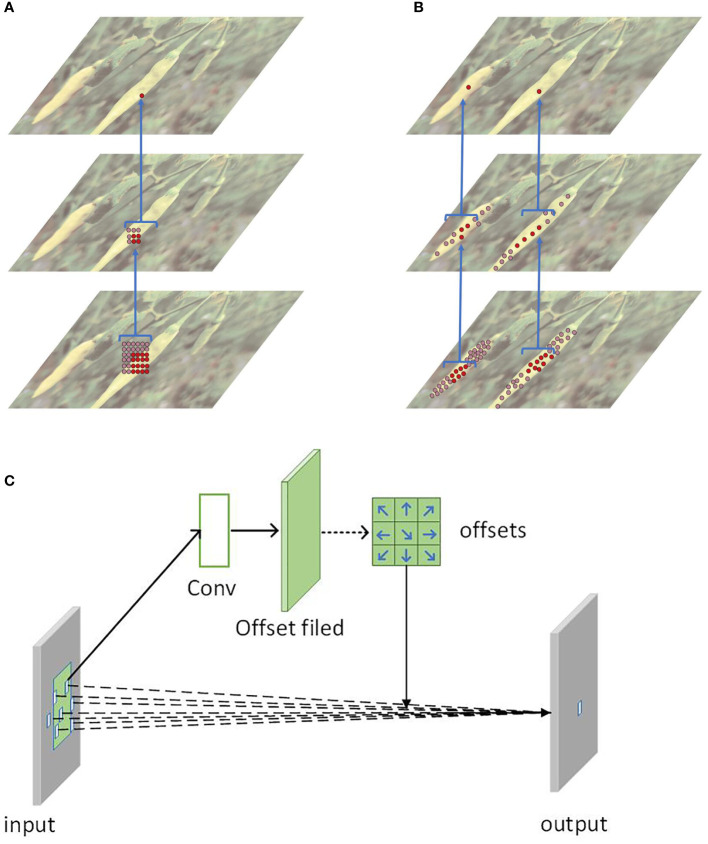
The deformable convolution module: **(A)** The traditional convolution method **(B)** The deformable convolution method **(C)** The principle of deformable convolution.

The backbone feature extraction network affects the parameters of YOLOv7, and its depth determines the speed of model detection. To meet the requirements of real-time detection, this study replaces the traditional convolution operation with the deformable convolution operation. The shape and size of each convolution kernel (filter) in the traditional convolution operation are pre-defined, and they cannot be changed during the convolution process, making it difficult to adapt to the shape change of the target. To solve the problem of the limited detection ability of traditional convolutional neural networks, Dai et al. (2017) proposed deformable convolution, as shown in **Figure 5**. The deformable convolution introduces a learnable deformable offset (deformable offset) so that the convolution kernel can be deformed with different shapes and spatial positions of the input data. By using deformable convolutions, the network can reduce the number of convolution kernels and parameters while maintaining the same receptive field size and the effectiveness of convolution operations. This is because the parameters of the deformable convolution are more compact than the traditional convolution; meanwhile, since the shape of the convolution kernel can be adaptively changed, it is more suitable for processing features of different shapes and positions. Thus, compared with traditional convolution, deformable convolution can reduce network parameters and improve the model’s ability to detect dense targets at different scales on the same plant.

**Figure 6:**
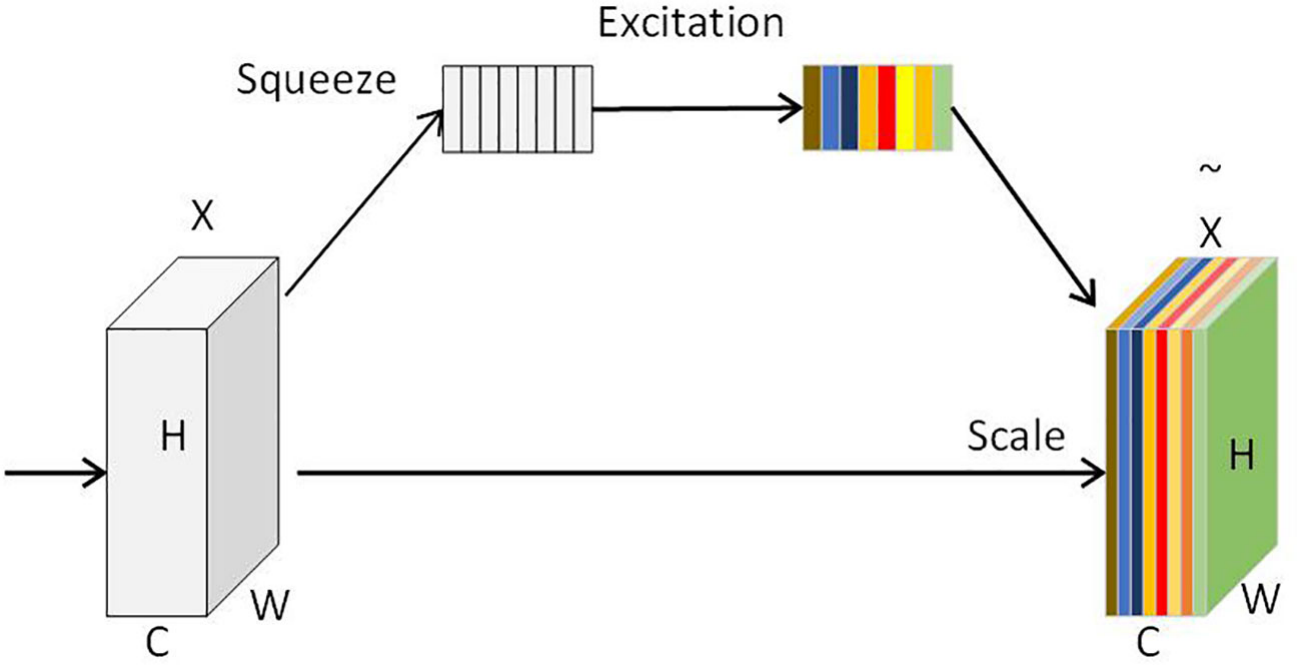
The structure of the SE module.

The attention mechanism was first proposed by MNIH (Zhang et al., 2022) and later introduced into the field of image classification. The visual attention mechanism embodies the visual characteristics of the human visual system that actively selects objects of interest and concentrates on them for processing. This characteristic can effectively improve image content screening, target retrieval, and image processing capabilities (Mnih et al., 2014). The attention mechanism is a technique used to improve the expressive power of neural network models. It guides the learning and prediction of the model by weighting different parts of input data, making the model focus more on the parts relevant to the task (Nan et al., 2023). This paper proposes to add the SE attention mechanism module (Dai et al., 2017) to the 14th and 21st layers of the backbone. This module (Hu et al., 2017) can adaptively adjust the channel weight of the feature map by learning a specific weight vector to improve the performance of the model. As illustrated in **Figure 6**, the SE attention mechanism includes two steps: the squeeze operation and the excitation operation. Specifically, the squeeze operation calculates the feature value of each channel through global average pooling. This process can compress the information of each channel into a value to obtain global information. The excitation operation uses a fully connected network layer to learn a non-linear function that takes as input the feature values of each channel from the previous step and outputs a new weight vector. Then, this new weight vector is scaled through a sigmoid activation function to assign attention weights to each channel. Compared with other attention mechanisms, the SE attention mechanism uses the global average pooling and fully connected layers, which are lightweight operations, so the SE module can improve the detection performance of such objects that are easily occluded without adding too much computational burden.

#### 2.3.3 The overall structure of the Xiaomila detection model

**Figure 7:**
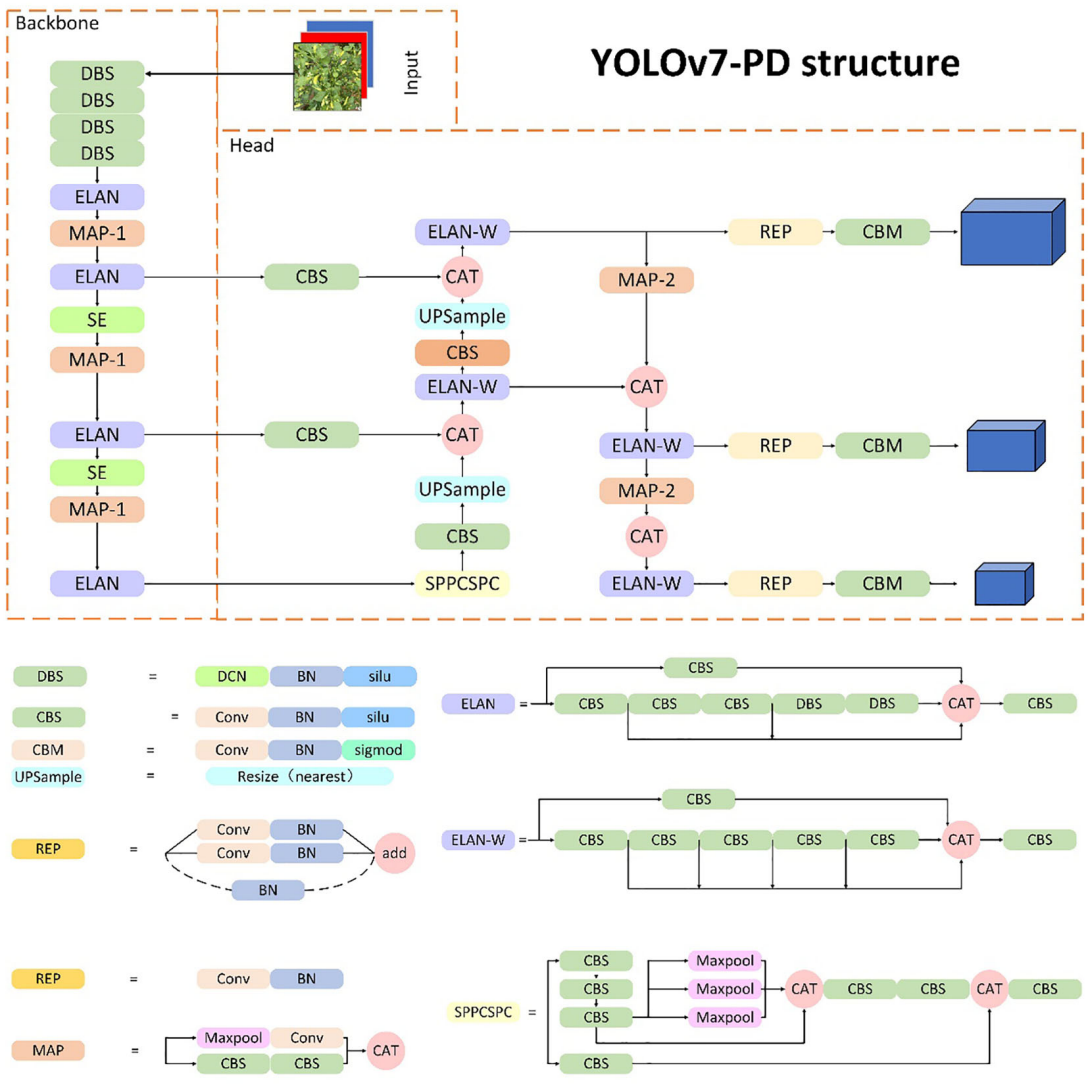
The structure of the YOLOv7-PD network.

Due to the high fruiting rate and irregular spatial distribution of Xiaomila plants, as well as the presence of Xiaomila targets of different scales on the same plant, it is difficult for most deep learning networks to accurately identify fruits in an unstructured environment. To address this issue, this paper replaces the 3×3 convolution kernel in the YOLOv7-tiny network skeleton with deformable convolution, and this is called deformable convolution (DCN). BN (batch normalization) and SiLU form the DBS module, which reduces the number of convolution kernels and parameters while maintaining the same receptive field as the traditional size convolution kernel. Meanwhile, the SE module is inserted in the 14th and 21st layers of the skeleton so that the model can learn the channel weight of the feature map of this layer while extracting features, which improves the detection ability of the model for small targets. The structure of the improved framework is shown in **Figure 7**. When an image is fed into the Xiaomila network, the YOLOv7-PD network initially resizes it to 640x640x3 before passing it through the backbone network. The feature maps are then processed by the DBS, ELAN, and Maxpooling modules, reducing their length and width by half while doubling the output channels. The MP module’s upper branch reduces the feature maps’ length and width by half via max pooling and their channels via convolution, while the lower branch halves the channels with the first convolution and reduces the feature maps’ length and width with the second convolution. The upper and lower branches are combined, producing a feature map with half the length and width and an equal number of input and output channels. The network assigns channel weights during feature extraction due to SE modules added at layers 14 and 21 in the backbone.Using the three-layer outputs of the backbone feature extraction network, the head network produces three different-sized feature maps. The final number of output channels is adjusted by the Repconv module before utilizing three 1x1 convolution layers for objectness, class, and bbox prediction tasks, yielding the Xiaomila detection outcomes.

### 2.4 Model training

#### 2.4.1 Training method and platform

The training platform is a desktop workstation equipped with 64 GB memory, an Intel Xeon^®^ W-214 CPU, and an NVIDIA RTX 2080Ti GPU (11 GB video memory). The operating system is Windows 11 (64-bit), the programming language is Python 3.9, the deep learning platform is CUDA 11.6, and the framework is Pytorch.

#### 2.4.2 Training strategy

In the model training process, the input image size was set to 640×640, the batch size was 16, the number of iterations was 300, the learning rate was set to 0.01, and the weight decay was set to 0.05. Since the Xiaomila detection method was proposed by changing the structure of the YOLOv7-tiny model, the pre-training weights provided by YOLOv7 cannot be used. Therefore, the YOLOv7-PD model proposed in this paper was not added with training weights at the training time, and the training data was saved in the model weight file to resume training in the case of interruption at the training time. Meanwhile, the training data of each iteration was saved for performance comparison and analysis of the model.

#### 2.4.3 Evaluation indicators

This paper adopted evaluation metrics including precision (P), recall (R), mean average precision (mAP), F1 score, detection speed, GFlops, and model weight.

mAP is the average precision of each class and the average value of AP, its calculation formula is:


$$mAP=\frac{1}{C}\int_{0}^{1}P\left(R\right)dR$$

The F1 score considers both precision and recall, and it can reflect the stability of a model. A higher F1 score indicates a more stable model. The formula for calculating the F1 score is:


$$F1=\frac{P\times R\times 2}{P+R}$$

P and R refer to the precision and recall of the detection model, respectively. Precision represents the proportion of true positive samples in the samples predicted as positive by the classifier. Recall represents the proportion of true positive samples that are correctly predicted as positive by the classifier among all true positive samples. The formula for calculating precision and recall is:


$$P=\frac{TP}{TP+FP}\times 100\%$$

$$R=\frac{TP}{TP+FN}\times 100\%$$

Detection speed refers to the number of image frames that the network model can detect per second. GFlops refers to the number of billions of floating-point operations performed per second, and it is used to evaluate the computational complexity of a network.

## 3 Results

### 3.1 Ablation experiment results

**Figure 8:**
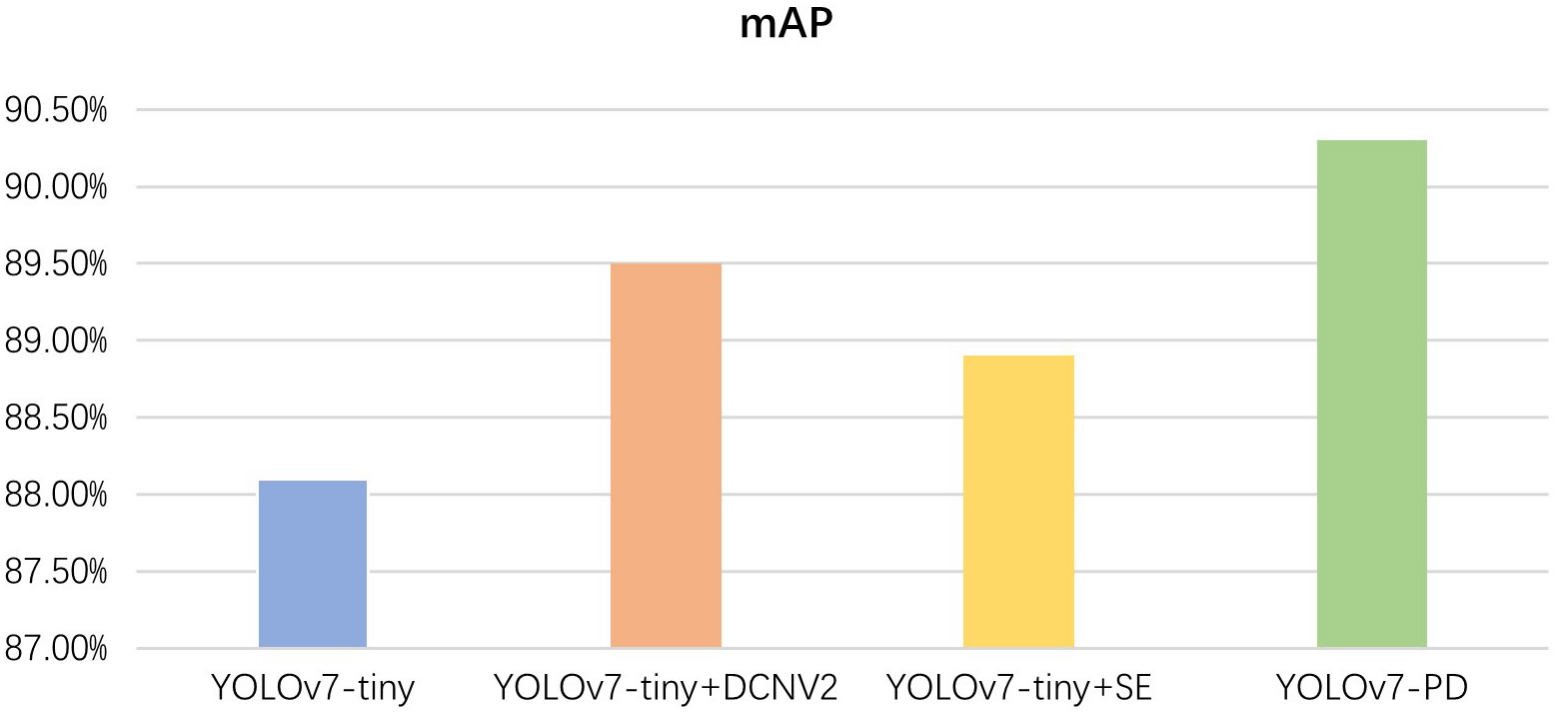
Ablation experiment results.

To investigate the impact of improved methods on detection accuracy, different improved models were tested, and ablation experiments were conducted. The test results are shown in **Figure 8**.

From the ablation experiment results, it can be seen that the proposed YOLOv7-PD model achieves the highest detection accuracy and the smallest model size.

### 3.2 Model comparison test results

To verify the advantages of the YOLOv7-PD model in detecting Xiaomila, this paper took three lightweight network models (Howard et al., 2019) including Mobilenetv3, YOLOv5s, and YOLOv7-tiny (Wang et al., 2022) for performance comparison. All deep learning detection algorithms adopted the same training and test datasets, and the input image size of the models was set to 640×640.

**Figure 9:**
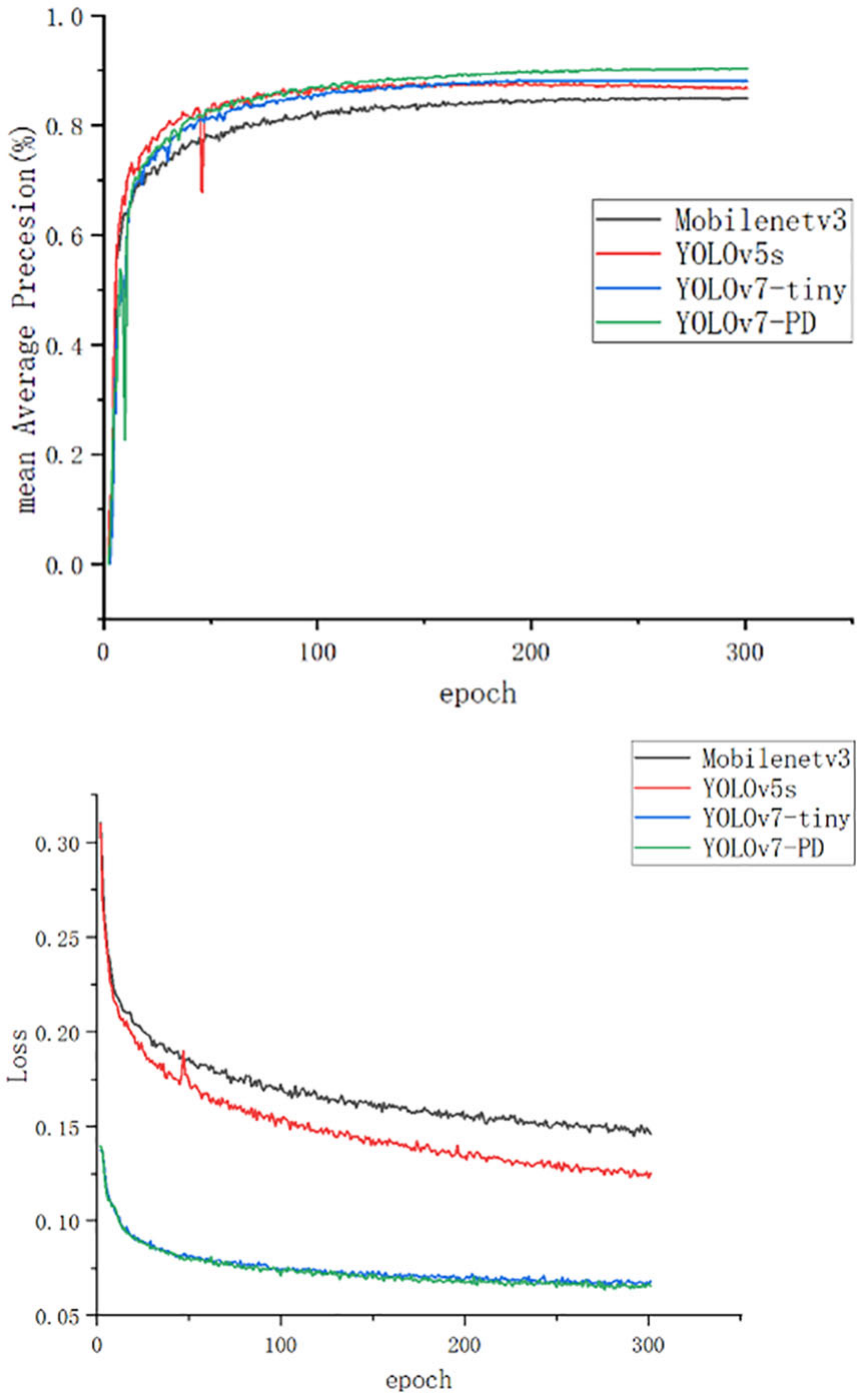
The mAP curve and loss curve.

**Figure 9** shows the mAP curve and loss curve of the models. Compared with the unimproved YOLOv7-tiny model, the improved YOLOv7-PD model converged faster and achieved higher accuracy. Affected by the addition of noise in the dataset, the mAP value of YOLOv5s began to decline after reaching the peak, while those of the other three models were not affected by the noise. During the training process of YOLOv7-tiny and YOLOv7-PD models, the model loss gradually stabilized when the number of iterations reached 100, and the final loss value tended to be stable at around 0.08, which was lower than that of Mobilenetv3 and YOLOv5s.

**Table 1** shows the comparison of each evaluation index between YOLOv7-PD and the other three deep-learning networks in the field detection of Xiaomila.

**Table 1:** The comparison of evaluation indices in the field detection.

Model	Precision	Recall	F1 Score	mAP	GFlops	Model Size
Mobilenetv3	84.1%	75.8%	79.95%	84.8%	11.3	10.1M
YOLOv5s	86%	77.9%	81.95%	86.7%	15.9	13.7M
YOLOv7-tiny	85.5%	81.3%	83.4	88.1%	13.1	12.7M
YOLOv7-PD	87.3%	81.3%	84.3%	90.3%	10.3	12.1M

It can be seen from **Table 1** that the mAP value of the YOLOv7-PD model was 90.3%, which was 2.2% higher than that of YOLOv7-tiny (88.1%), 9.9% higher than that of Mobilenetv3 (80.4%), and 3.3% higher than that of YOLOv5s (87%). The experimental results indicated that compared to other models, YOLOv7 has advantages in all aspects. The model size of YOLOv7-PD is 12.1 MB, and the number of calculations per second is 10.3 GFlops. Compared with YOLOv7-tiny and YOLOv5s, both the number of computations per unit of time and the model size have been reduced. Compared with Mobilenetv3, although the model size has increased, the speed and accuracy of the model have been improved.

By analyzing the experimental results, the YOLOv7-PD model reduces the training time and model size while improving the detection accuracy, contributing to a lightweight detection model. The model is significantly superior to the other three deep learning networks in terms of model parameters, weights, GFlops, etc., indicating that it is more suitable for deployment on agricultural mobile devices.

### 3.3 Comparison of model detection effects

**Figure 10:**
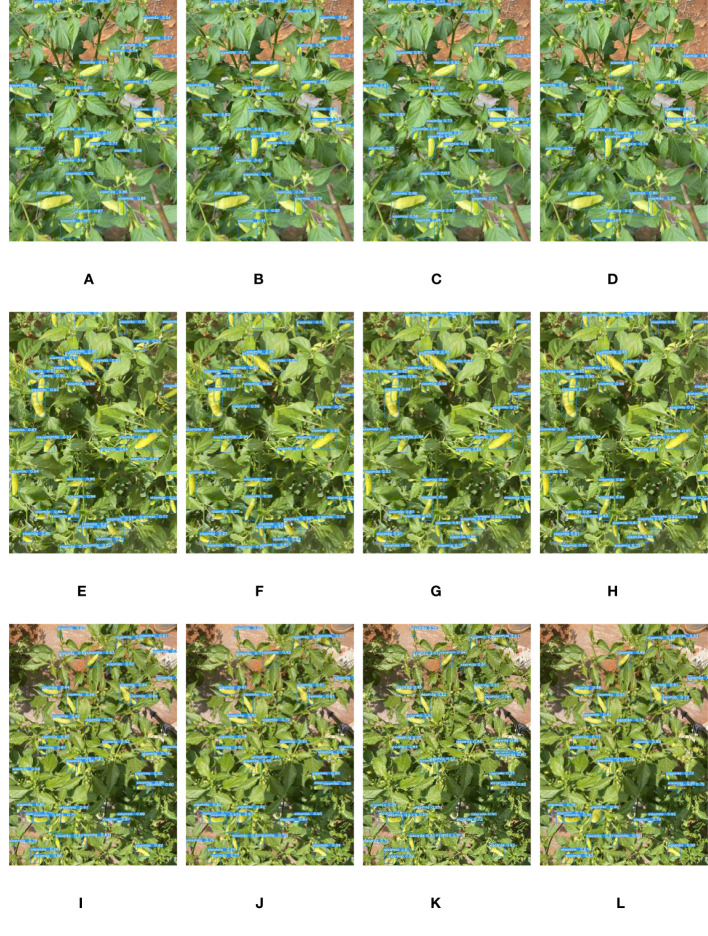
Comparison of detection results of each model under weak, medium, and strong light intensity: **(A, E, I)** YOLOv7-PD; **(B, F, J)** YOLOv7-tiny; **(C, G, K)** YOLOv5s; **(D, H, L)** Mobilenetv3.

To verify the Xiaomila detection performance of YOLOv7-PD, YOLOv7-tiny, YOLOv5s, and Mobilenetv3 models, 90 Xiaomila images under different lighting conditions in the test set were used for testing. Among these images, 33 images have strong light intensity and include 639 Xiaomila peppers, 28 images have medium light intensity and include 491 Xiaomila peppers, and 29 images have weak light intensity and include 582 Xiaomila peppers. The test results are shown in **Table 2** and **Figure 10**.

**Table 2:** Xiaomila detection results under different lighting conditions.

Lighting conditions	Model	Quantity	The number of correct detections	The number of false detections	The number of failed detections
strong light intensity	YOLOv7-PD	639	562	72	77
	YOLOv7-tiny	639	559	83	80
	YOLOv5s	639	550	110	89
	Mobilenetv3	639	524	132	115
low light intensity	YOLOv7-PD	491	428	55	63
	YOLOv7-tiny	491	423	71	68
	YOLOv5s	491	426	63	65
	Mobilenetv3	491	410	78	81
backlight	YOLOv7-PD	582	527	49	55
	YOLOv7-tiny	582	509	58	73
	YOLOv5s	582	501	61	81
	Mobilenetv3	582	479	53	103

Overall, in the case of weak light intensity, the detection difficulty increased, and in the case of strong light intensity, the characteristics of the object were easier to learn by the model, and most fruits can be recognized. Specifically, the YOLOv7-PD model proposed in this paper showed better performance. The performance of YOLOv7-tiny was similar to that of YOLOv7-PD, which was much higher than that of YOLOv5s, and Mobilenetv3 obtained the worst performance. The results indicate that YOLOv7-PD can still accurately recognize Xiaomila under different lighting conditions.

**Figure 11:**
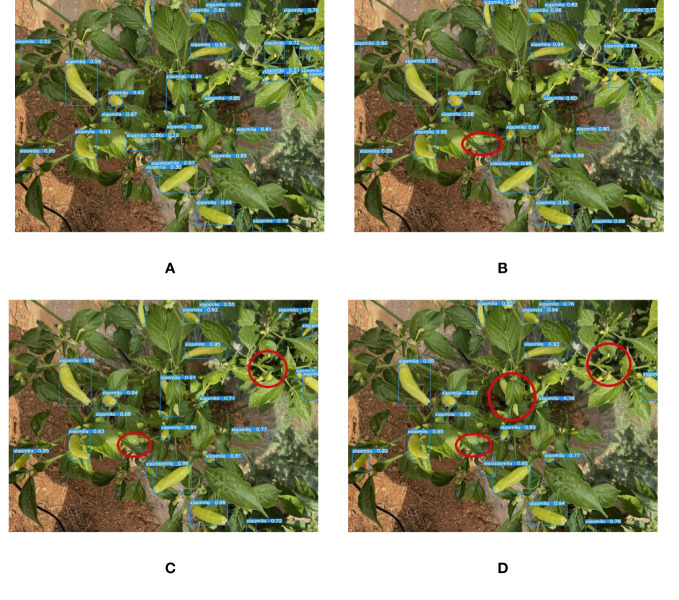
The detection of occluded targets by each model. **(A)** YOLOv7-PD; **(B)** YOLOv7-tiny; **(C)** YOLOv5s; **(D)** Mobilenetv3.

Detecting Xiaomila fruits on the Xiaomila plant in the field environment and the occlusion problem has become the key issues to address in the research. The current deep learning models are difficult to accurately identify the occluded target, but the YOLOv7-PD model proposed in this paper can detect some occluded Xiaomila fruits, and the detection effect is shown in **Figure 11**. Compared with other models, the YOLOv7-PD model significantly improves the detection ability of fruits occluded by branches and leaves.

**Figure 12:**
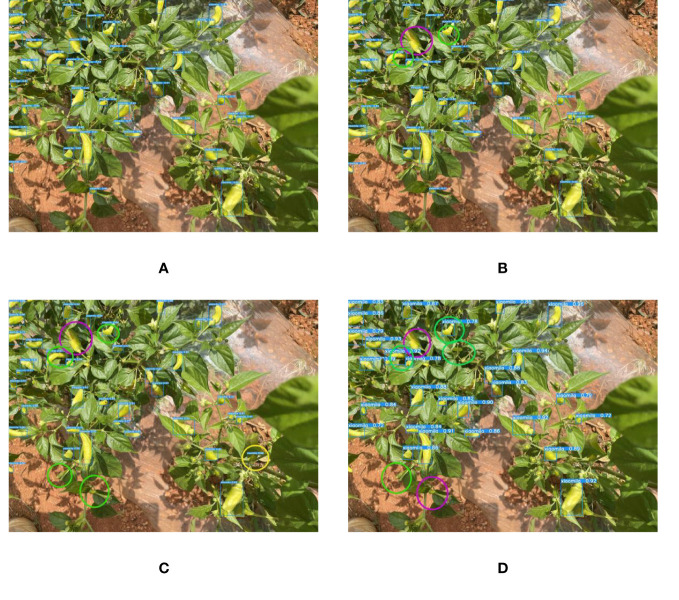
The detection results of each model in detecting objects with similar colors to fruits and branches and leaves. **(A)** YOLOv7-PD; **(B)** YOLOv7-tiny; **(C)** YOLOv5s; **(D)** Mobilenetv3.

Since the color of the Xiaomila fruit in the green and ripe periods is similar to that of the stems and leaves of the plant, other models suffer from the problem of misidentifying leaves as fruits and missing the detection of fruits. The proposed YOLOv7-PD model shows good performance in solving the problem of poor detection of target fruits with similar colors, as shown in **Figure 12**, In the figure, purple circles indicate missed detections caused by similar colors, yellow circles indicate false detections caused by similar colors, and green circles indicate missed detections caused by dense fruit. YOLOv7-PD demonstrates better detection performance on occluded targets, while YOLOv7-tiny struggles to detect partially occluded targets with larger areas. Additionally, YOLOv5s misses the detection of two occluded fruits, while Mobilenetv3 performs poorly in detecting occluded targets.

Xiaomila takes more than a year to ripen, and the nature of the same period of flowers and fruits leads to a high fruit-setting rate per plant. There are objects of different scales on the same plant, and the distribution of each object is very close. For detecting large-scale Xiaomila targets, each model can perform well. However, for the alternate distribution of Xiaomila targets of different scales, each model suffers from the problem of missed detection or false detection.

**Figure 13:**
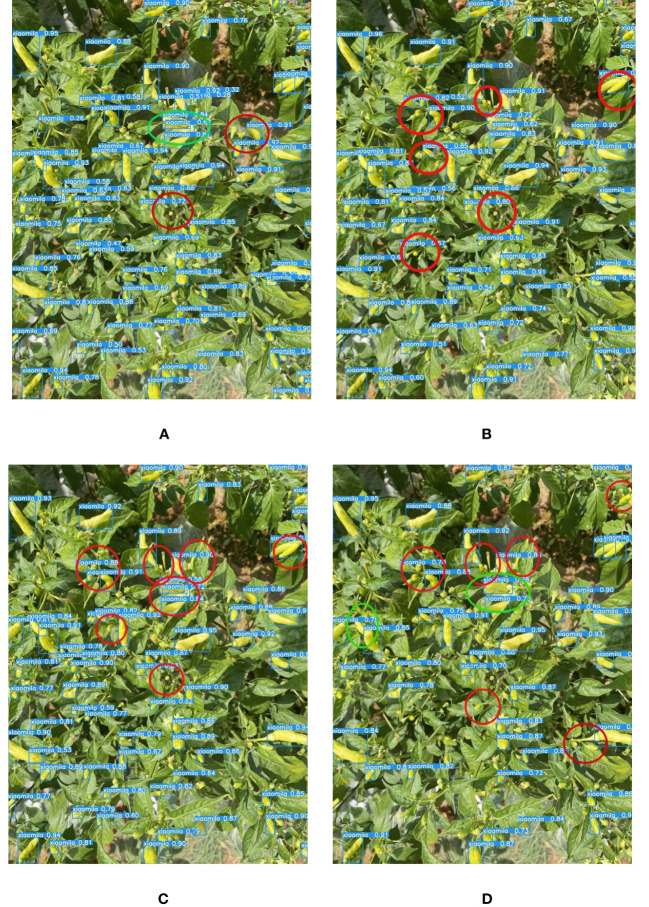
The detection of dense multi-scale targets by each model. **(A)** YOLOv7-PD; **(B)** YOLOv7-tiny; **(C)** YOLOv5s; **(D)** Mobilenetv3.

It can be seen from **Figure 13** that although YOLOv7-PD mis-detects and misses a small number of small targets, it shows the best performance compared to the other three models. The figure highlights missed and false detections made by the model. The green circles in the figure shows the misdetection of small targets, and the red circles shows the missed detection of small targets: It not only ensures the detection accuracy of Xiaomila but also reduces the calculation amount per unit time of the model and the size of the model weight file; besides, it can identify dense targets, small targets, occluded targets, and targets with similar colors to branches and leaves. The above comparative experiments indicate that the YOLOv7-PD model proposed in this study has certain advantages in detection speed and detection accuracy. Overall, it can accurately identify Xiaomila under complex lighting and background conditions, laying the foundation for Xiaomila’s automatic picking.

## 4 Discussion

Compared to other single-stage models, the proposed YOLOv7-PD model achieves better performance by reducing the model weight files and improving the detection accuracy of occluded and alternatively distributed targets in complex environments while increasing the computational speed. To further validate the effectiveness of the model, it was trained on our dataset using other improved deep learning detection methods (Li et al., 2021; Bosquet et al., 2023; Nan et al., 2023) mentioned in this paper, and the detection results on the Xiaomila dataset under different illuminations were compared, as shown in **Tables 3**, **4**.

**Table 3:** Comparison of training results.

Model	Precision	Recall	F1	mAP	GFlops	Model Size
YOLOv7-PD	87.3%	81.3%	84.3%	90.3%	10.3	12.1M
YOLOv4-tiny (Li et al., 2021)	85.9%	78.9%	82.4%	86.8%	48.2	30.9M
YOLOv5l (Nan et al., 2023)	86.2%	79.9%	83.05%	87.2%	41	22.3M

**Table 4:** Comparison of detection results.

Lighting conditions	Model	Quantity	The number of correct detections	The number of false detections	The number of missed detections
strong light intensity	YOLOv7-PD	639	562	72	77
	YOLOv4-tiny (Li et al., 2021)	639	553	70	86
	YOLOv5l (Nan et al., 2023)	639	559	92	80
low light intensity	YOLOv7-PD	491	428	55	63
	YOLOv4-tiny (Li et al., 2021)	491	420	63	71
	YOLOv5l (Nan et al., 2023)	491	427	61	64
backlight	YOLOv7-PD	582	527	49	55
	YOLOv4-tiny (Li et al., 2021)	582	499	55	83
	YOLOv5l (Nan et al., 2023)	582	513	47	71

The results indicate that compared to other pepper detection models, the YOLOv7-PD model proposed in this paper has advantages in both model size and detection accuracy.

## 5 Conclusions

This paper proposes a method for the detection of Xiaomila fruits in complex field environments. In this method, YOLOv7-tiny is selected as the transfer learning model for field detection of Xiaomila fruits. Meanwhile, the backbone extraction network is integrated with deformable convolution, the DCN is used to replace the YOLOv7-tiny backbone and the traditional convolution module in the ELAN module, and the network’s ability to extract multi-scale target features is improved. Besides, the SE attention mechanism is inserted into the reconstructed backbone feature extraction network to improve its ability to extract the key features of Xiaomila peppers and realize multi-scale Xiaomila pepper fruit detection in complex environments. Moreover, the detection performance of three other single-stage object detection networks is compared and analyzed. Through the analysis of the experimental results, it can be seen that the improved model significantly enhances the detection effect of dense multi-scale targets while reducing the model training parameters and improving the detection speed. It has achieved excellent performance on the Xiaomila dataset with complex backgrounds and different lighting conditions.

There are certain limitations to this study because the algorithm proposed in this article can only recognize the Xiaomila fruit in the image, but in practical applications, we not only need to recognize the Xiaomila fruit but also locate it. In future work, we will concentrate on the detection of Xiaomila picking points and the determination of Xiaomila’s growth direction with a depth camera and migrate the detection model to the embedded device to realize the automatic picking of Xiaomila.

## Data availability statement

The raw data supporting the conclusions of this article will be made available by the authors, without undue reservation.

## Author contributions

JJ designed the lightweight network and trained the model. YC, ZS, and HZ collected the Xiaomila images. YT performed the image calibration. FW revised the manuscript. QL provided guidance for the experiments. All authors contributed to the article and approved the submitted version.
